# JAM-A knockdown accelerates the proliferation and migration of human keratinocytes, and improves wound healing in rats via FAK/Erk signaling

**DOI:** 10.1038/s41419-018-0941-y

**Published:** 2018-08-28

**Authors:** Yunchuan Wang, Jianping Zheng, Yue Han, Yijie Zhang, Linlin Su, Dahai Hu, Xiaobing Fu

**Affiliations:** 1 Institute of Basic Medicine, Chinese PLA General Hospital, 100853 Beijing, China; 2 Department of Burns and Cutaneous Surgery, Xijing Hospital, The Fourth Military Medical University, 710032 Xi’an, Shaanxi China; 3 Department of Orthopedic Surgery, Xiangyang Central Hospital, The Affiliated Hospital of Hubei University of Arts and Science, Xiangyang, 441021 Hubei China; 4 Department of Burns and Plastic Surgery, Xi’an Central Hospital, 710003 Xi’an, Shaanxi China

## Abstract

Junctional adhesion molecule-A (JAM-A) belongs to the immunoglobulin superfamily, it predominantly exists at the tight junctions of epithelial and endothelial cells. JAM-A is known to regulate leukocyte trans-endothelial migration, however, how it affects the proliferation and migration of keratinocytes, the two essential steps during wound healing, has less been explored. In this study, we showed that JAM-A was significantly expressed in normal skin epidermis. RNAi-mediated JAM-A knockdown remarkably promoted the proliferation and migration of keratinocytes. We also found that loss of JAM-A increased the protein levels of *p*-FAK, *p*-Erk1/2, and *p*-JNK; however, FAK inhibitor PF-562271 restrained the expression of *p*-FAK and *p*-Erk1/2 elevated by JAM-A RNAi, but not *p*-JNK, and also slowed down keratinocyte proliferation and migration. Finally, in a rat wound model we showed that absence of JAM-A significantly promoted the wound healing process, while the use of PF-562271 or Erk1/2 inhibitor PD98059 repressed those effects. These data collectively demonstrate that suppressing JAM-A expression could promote the proliferation and migration of keratinocytes and accelerate the healing process of rat skin wounds, potentially via FAK/Erk pathway, indicating that JAM-A might serve as a potential therapeutic target for the treatment of chronic refractory wounds.

## Introduction

The process of skin wound healing is well organized by multiple factors. Re-epithelialization, a key step during wound healing, relies on two essential behaviors of keratinocytes: proliferation, and migration^1^. Junctional adhesion molecule-A (JAM-A, previously referred as JAM-1) is a 36-kD trans-membrane molecule and specifically locates at tight junctions, which are formed between two adjacent epithelial or endothelial cells^2^. JAM-A has been implicated to participate in leukocyte migration across the endothelium^3^, potentially via mitogen-activated protein kinase (MAPK) and/or focal adhesion kinase (FAK)-mediated pathways, which are necessary for migration^4^. However, it is unknown whether JAM-A takes part in keratinocyte migration and cutaneous wound healing, and if so, which signaling is involved in these processes.

JAM-A belongs to the immunoglobulin superfamily of adhesion molecules, it is found in platelets^5^, endothelial and epithelial cells, and most leukocyte subsets^6^. JAM-A is shown to play multi-functional roles in various of cellular physiological activities. For instance, JAM-A engaged in the formation of tight junctions and helps maintain the integrity of epithelia and endothelia^6,7^; JAM-A was found to accept certain sub-strains of the reovirus and promotes their invasion into cells^8^; JAM-A was show to act as a ligand during leukocyte migration across the endothelia^3^. Moreover, a study has clearly shown that JAM-A deficiency boosted the spontaneous and random motility in mouse endothelial cells^9^. Additionally, when JAM-A was suppressed, the proliferation of endothelial cells and the formation of capillary vessels were remarkably retarded^10^. In contrast, overexpression of JAM-A was found to strengthen cell–cell interaction and thus suppress the migration of endothelial cells^11^. As a crucial component at tight junction, JAM-A is likely to be involved in keratinocyte proliferation and migration, as well as cutaneous wound healing.

Focal adhesion kinase (FAK), best known as a non-receptor cytoplasmic tyrosine kinase, exerts essential roles in diversities of cellular behaviors such as survival, proliferation, and migration. When FAK’s activity was inhibited through overexpressing FAK C-terminal, a significant decrease in the proliferation of HUVECs was noticed^12^. FAK was found to be significantly activated and phosphorylated in epithelial and endothelial cells during wound healing process, suggesting a functional role of FAK in cell migration^13^. FAK regulates many signaling pathways, such as PI3K/Akt, mTOR/STAT3, and Ras activation, that are closely related to cell proliferation and migration. The well-known MAPK molecules, namely Erk1/2, p38 MAPK and JNK, are actively involved in many cellular behaviors, including cell proliferation and migration^14^, while FAK is an important activator of MAPK signaling^15^.

Therefore, the current study proposed that JAM-A might play a role in the proliferation and migration of keratinocytes, and also affect the skin healing process, partially through the FAK-MAPK signaling. To investigate these aspects, we conducted a series of experiments with two models: (1) the primary human keratinocytes, and (2) the rats with dorsal skin incision. Next, JAM-A knockdown by RNAi was performed to investigate the regulative mechanism show JAM-A regulates keratinocyte proliferation and migration, as well as in vivo wound healing. Two drugs, PF-562271 and PD98059, were used to inhibit FAK activity^16^ and Erk1/2 activity^17^, respectively. Our data demonstrate that the deficiency of JAM-A expression stimulated the proliferation and migration of human keratinocytes and improved the in vivo wound healing in rats, potentially through FAK/Erk signaling.

## Results

### JAM-A predominantly exists in the epidermis of normal human skin

**Fig. 1:**
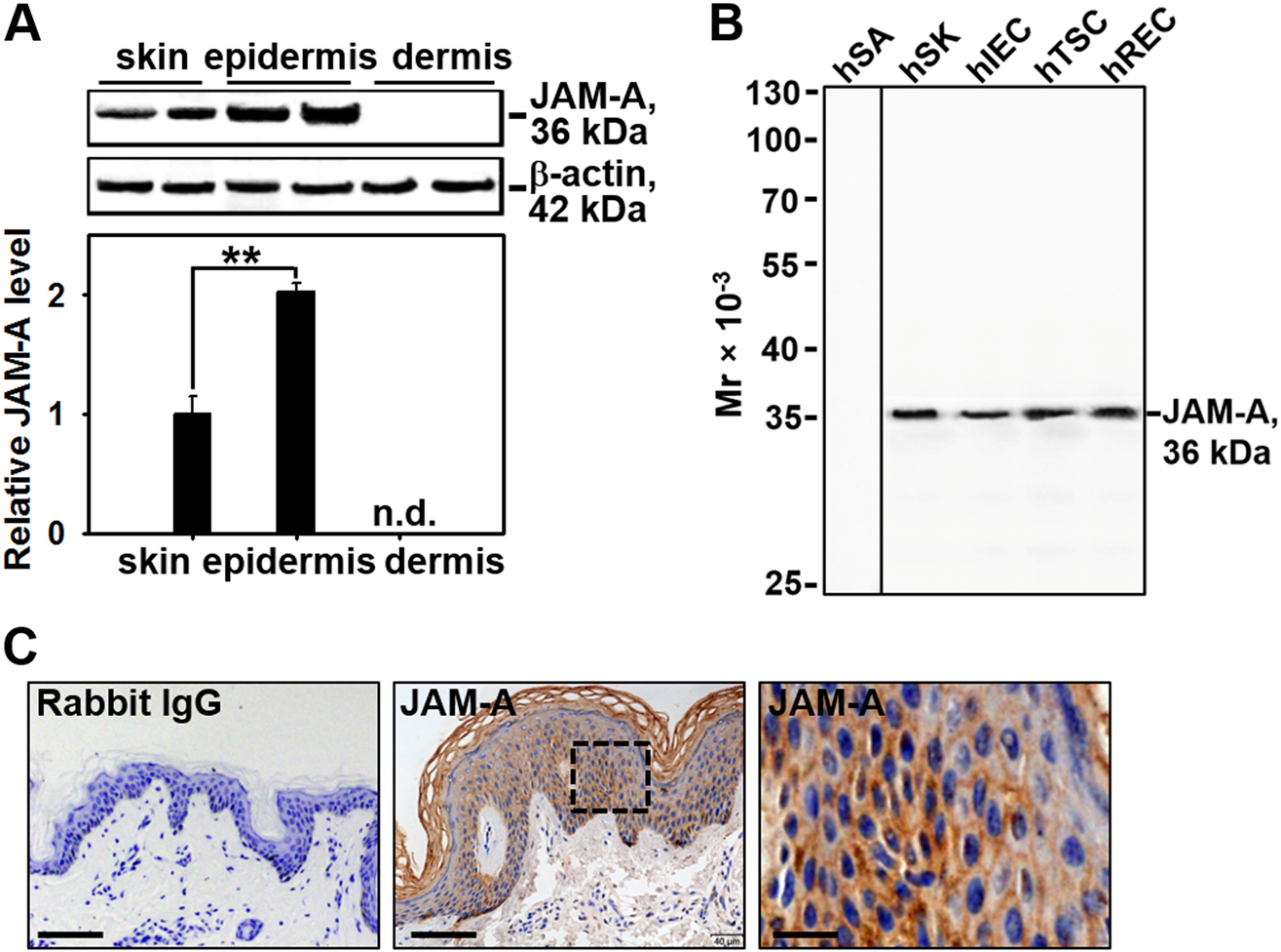
JAM-A expression and distribution in human skin tissues. **a** Western blot analysis comparing the difference of JAM-A expression among the full-thickness skin, epidermis, and dermis. The protein level of actin was used herein as the equal loading control. JAM-A expression in the epidermis or dermis was compared to that in the skin, which was designated as 1 after normalization against actin. Each bar was the mean ± SD using 3–4 skin samples from different individuals. **P < 0.01; n.d. not detectable. **b** The immunoblot showing the specificity of JAM-A antibody used in this study with lysates from human skin keratinocytes (hSK), intestinal epithelial cells (hIEC), testicular Sertoli cells (hTSC), and retinal endothelial cells (hREC). **c** Representative images from paraffin sections of human skin tissues stained with anti-JAM-A antibody by IHC. JAM-A-specific staining appeared as brownish precipitates as seen in the middle image, scale bar = 60 µm. The right image is the corresponding magnified boxed area shown in the middle, bar = 12 µm. Anti-JAM-A antibody was replaced with normal rabbit IgG as the negative control shown in the left image, bar = 60 µm

It is known that JAM-A could regulate leukocyte trans-endothelial migration, we are curious about if JAM-A plays a role during the healing process. The protein level of JAM-A was first compared among the full-thickness skin, the epidermis, and the dermis from normal human skin by immunoblotting with a specific JAM-A primary antibody (see Supplementary Table S2). Results showed that the expression level of JAM-A in epidermis was twofolds more than JAM-A level of the full-thickness skin, dermis expressed undetectable level of JAM-A (Fig. 1a). Also, lysates from human skin keratinocytes (hSK)^18^, intestinal epithelial cells (hIEC)^19^, testicular Sertoli cells (hTSC)^20^, and retinal endothelial cells (hREC)^21^ were used to assess the specificity of this JAM-A antibody by western blot. Results showed that all the four types of cells expressed JAM-A at a moderate level, while human subcutaneous adipocytes (hSA) did not express JAM-A and served as the negative control. Therefore, this anti-JAM-A antibody is ideal for subsequent experiments due to its specificity (Fig. 1b). Immunohistochemistry (IHC) staining on paraffin sections of normal human skin showed that JAM-A predominantly located at the epidermal part of the skin, and concentrated at cell–cell interface, which coincides with previous reports that JAM-A is a putative tight junction protein, while JAM-A was absent in the dermis (Fig. 1c). IHC results are in accordance with the findings by western blot in Fig. 1a. Above data reveal that JAM-A predominantly expresses at the epidermis of normal human skin.

### JAM-A knockdown by RNAi promotes the proliferation and migration of normal human keratinocytes

**Fig. 2:**
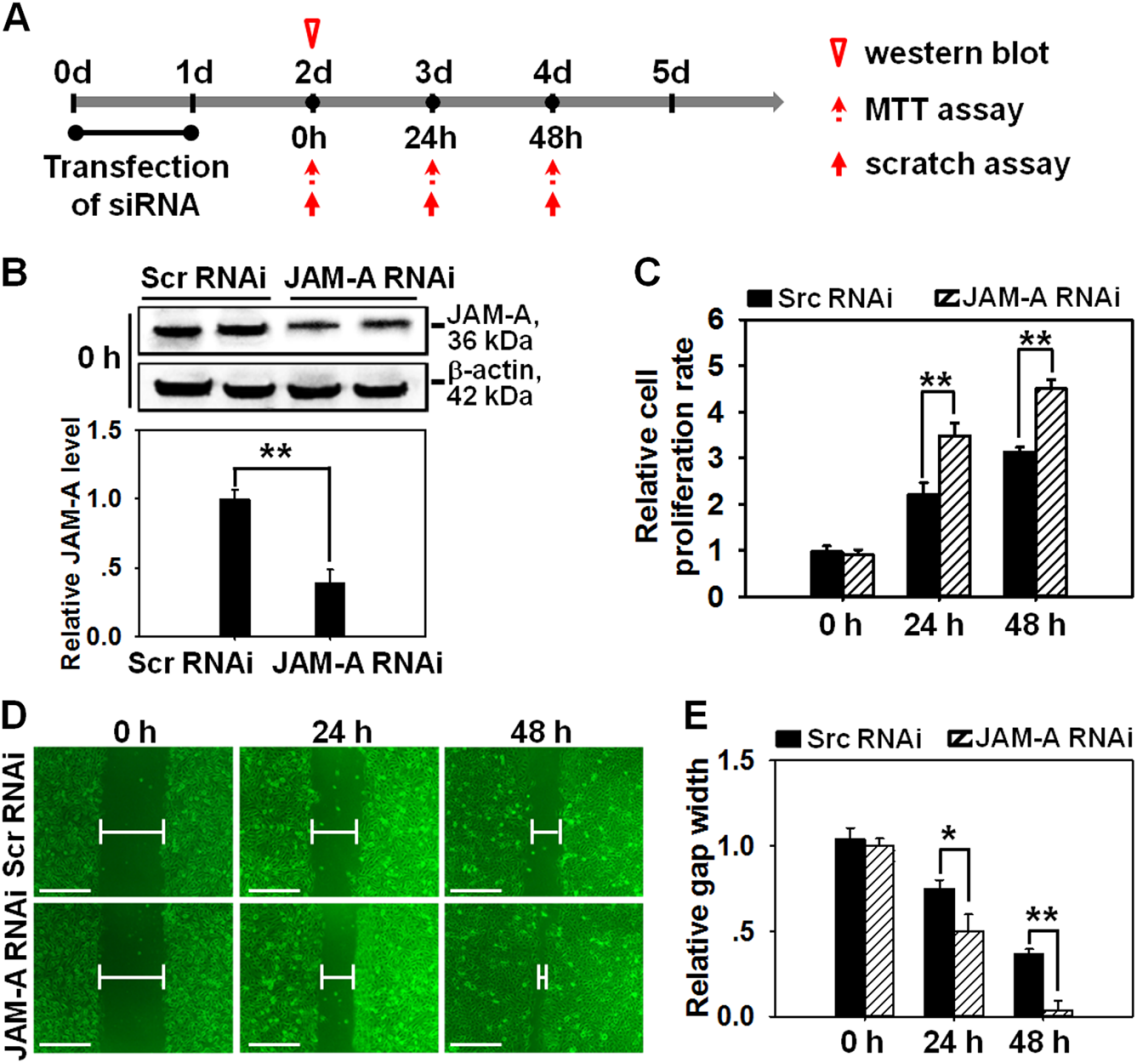
Effect of JAM-A silencing on the proliferation and migration of primary keratinocytes. **a** Time points selected for the in vitro study. Generally, siRNA duplexes for scramble (Scr) or JAM-A were transfected into keratinocyte cultures on day 0. After 24-h reaction, the mixture was replaced with fresh medium, cells were then cultured for another 24 h followed by different assays to examine the protein expression, cell proliferation, and migration at appointed times. **b** Western blot showing an ~70% knockdown of JAM-A expression in keratinocytes 2 days after RNAi treatment. Densitometric analysis of JAM-A immunoblotting data normalized against actin with scramble RNAi arbitrarily set at 1. Each bar represents mean ± SD from four experiments. **P < 0.01. **c** The proliferation of keratinocytes after JAM-A silencing was evaluated by MTT assay at 0, 24, 48 h. Bars represent the mean ± SD, *n* = 4. **d** The migration of keratinocytes after JAM-A silencing was evaluated by cell scratch assay. Notably, keratinocytes were pre-treated with mitomycin C for 1 h before scratch assay. Images at 0, 24, and 48 h post-scratching were captured to display the process of gap closure. Scale bars = 100 µm. **e** Densitometric analysis of the data from scratch assay normalized against the gap length at 0 h in scramble RNAi group, which was designated as 1. Bars are means ± SD, *n* = 4. *P < 0.05; **P < 0.01

The appropriate proliferation and migration of skin epithelial cells are critical for normal healing process, thus the regulatory effects of JAM-A on cell proliferation and migration were then investigated. RNAi technique was conducted to specifically knockdown JAM-A expression. Briefly, primary keratinocytes were transfected by RNAi for 24 h from day 0 to day 1, followed by immunoblotting, cell proliferation assay, and cell scratch to investigate JAM-A expression, keratinocyte proliferation and migration at selected time points from day 2 to day 4 (Fig. 2a). Immunoblots displayed a decrease on JAM-A level by 70% after JAM-A knockdown on day 2 at 0 h (i.e., one day after the completion of RNAi transfection) (Fig. 2b). The efficiency and non-off target effect of JAM-A siRNA duplexes used in this study were also confirmed by real-time PCR and western blot analyses. Results showed that both JAM-A siRNA sequences #1 and #2 could significantly suppress JAM-A level by 70–80%, while neither of them affected the mRNA and protein levels of the selected cell junction molecules, such as occludin (a tight junction protein), claudin-11 (a tight junction protein), E-cadherin (an adhesion junction protein), ZO-1 (a tight junction adaptor), desmoglein-2 (a desmosome junction protein), α6-integrin (a hemi-desmosome junction protein), and β4-integrin (a hemi-desmosome junction protein) (see Supplemental Fig. S1 and S2). MTT assay showed increased cell proliferation rate in JAM-A RNAi-transfected keratinocytes at 24 h and 48 h (i.e., on day 3 and day 4) compared to that in scramble RNAi group (Fig. 2c). To ignore the influence from cell proliferation and observe the net result of JAM-A RNAi on migration, keratinocytes were pre-treated with mitomycin C for 1 h in cell scratch assay. Images showed that JAM-A knockdown remarkably promoted the movement of keratinocytes and reduced the gap length at 24 and 48 h after scratching (i.e., on day 3 and day 4) compared to scramble RNAi group (Fig. 2d, e). Notably, the scratch gap in JAM-A knockdown cells was almost fully closed at 48 h post-scratching (Fig. 2d, e). These results suggest that the proliferation and migration of keratinocytes could be enhanced through the suppression of JAM-A expression.

### JAM-A knockdown by RNAi induces the phosphorylation of FAK, ERK1/2, and JNK in primary human keratinocytes

**Fig. 3:**
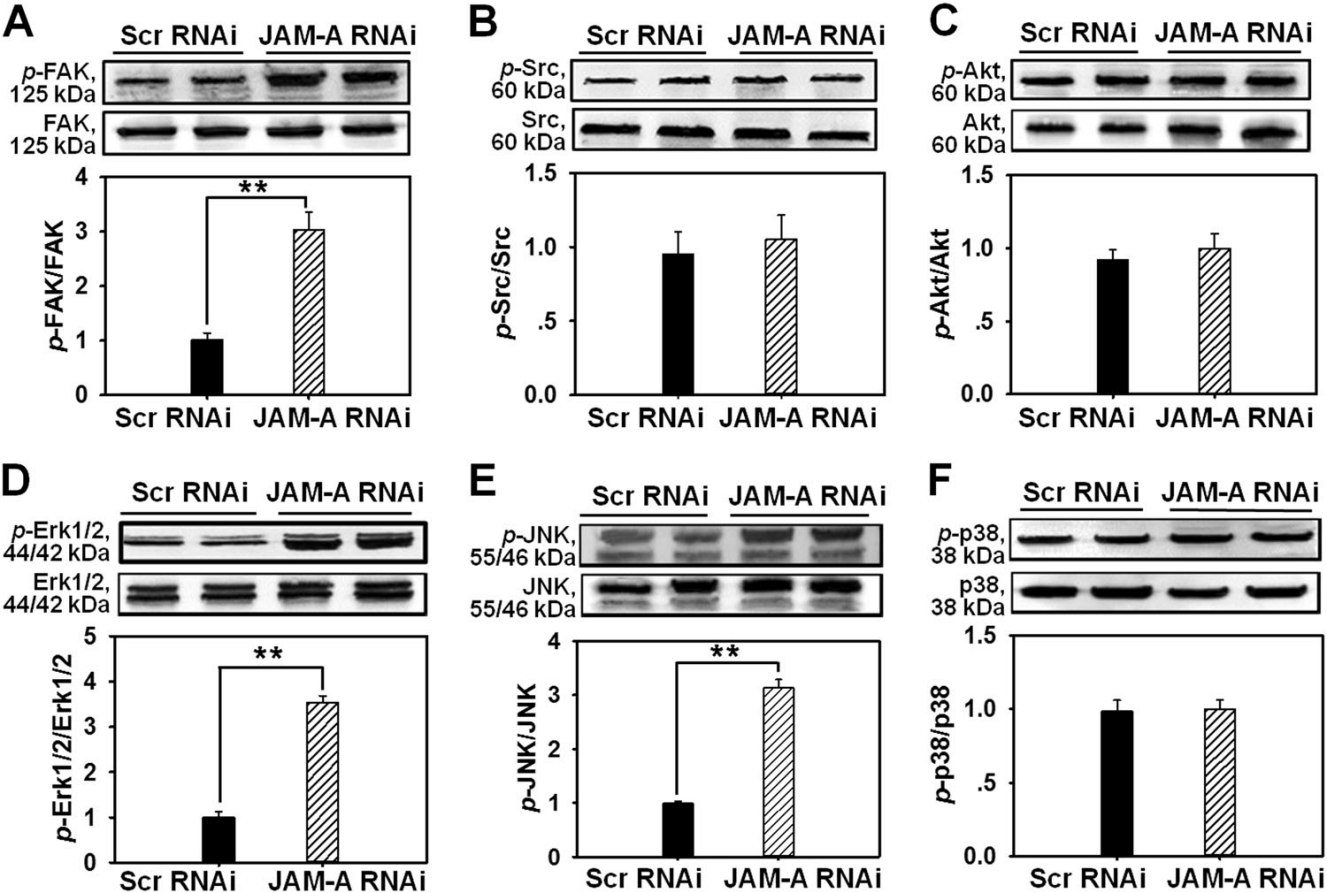
Changes on the protein levels of selected signaling molecules after JAM-A silencing in primary keratinocytes. Western blot analysis compared the expression of the total FAK (**a**), Src (**b**), Akt (**c**), Erk1/2 (**d**), JNK (**e**), p38 (**f**), and their corresponding phosphorylated forms in keratinocytes before and after JAM-A knockdown. Densitometric analysis on the levels of phosphorylated proteins were normalized against the corresponding total protein levels, which was then designated as 1. Bars are the means ± SD, *n* = 4. **P < 0.01

In order to study the mechanism of JAM-A knockdown-induced enhancement of cell proliferation and migration, we then examined the change on protein levels of two tyrosine kinases FAK and Src, Akt, three MAPKs Erk1/2, JNK and p38, as well as their phosphorylated proteins after JAM-A RNAi in primary human keratinocytes. Immunoblots showed that the total FAK, Src, Akt, Erk1/2, JNK or p38 level remained stable before and after RNAi (Fig. 3a–f). However, the level of phosphorylated FAK (Fig. 3a), phosphorylated Erk1/2 (Fig. 3d), or phosphorylated JNK (Fig. 3e) remarkably raised by 2.9, 3.4, or 3.1 times, respectively, while *p*-Src (Fig. 3b), *p*-Akt (Fig. 3c), or *p*-p38 (Fig. 3f) level was not affected by RNAi, indicating that FAK, Erk1/2, and JNK were activated after the loss of JAM-A. Above data demonstrate that FAK, Erk1/2, and JNK might be involved in JAM-A knockdown-induced enhancement of keratinocyte proliferation and migration.

### The inhibition of FAK by PF-562271 differentially affects Erk1/2 and JNK signaling

**Fig. 4:**
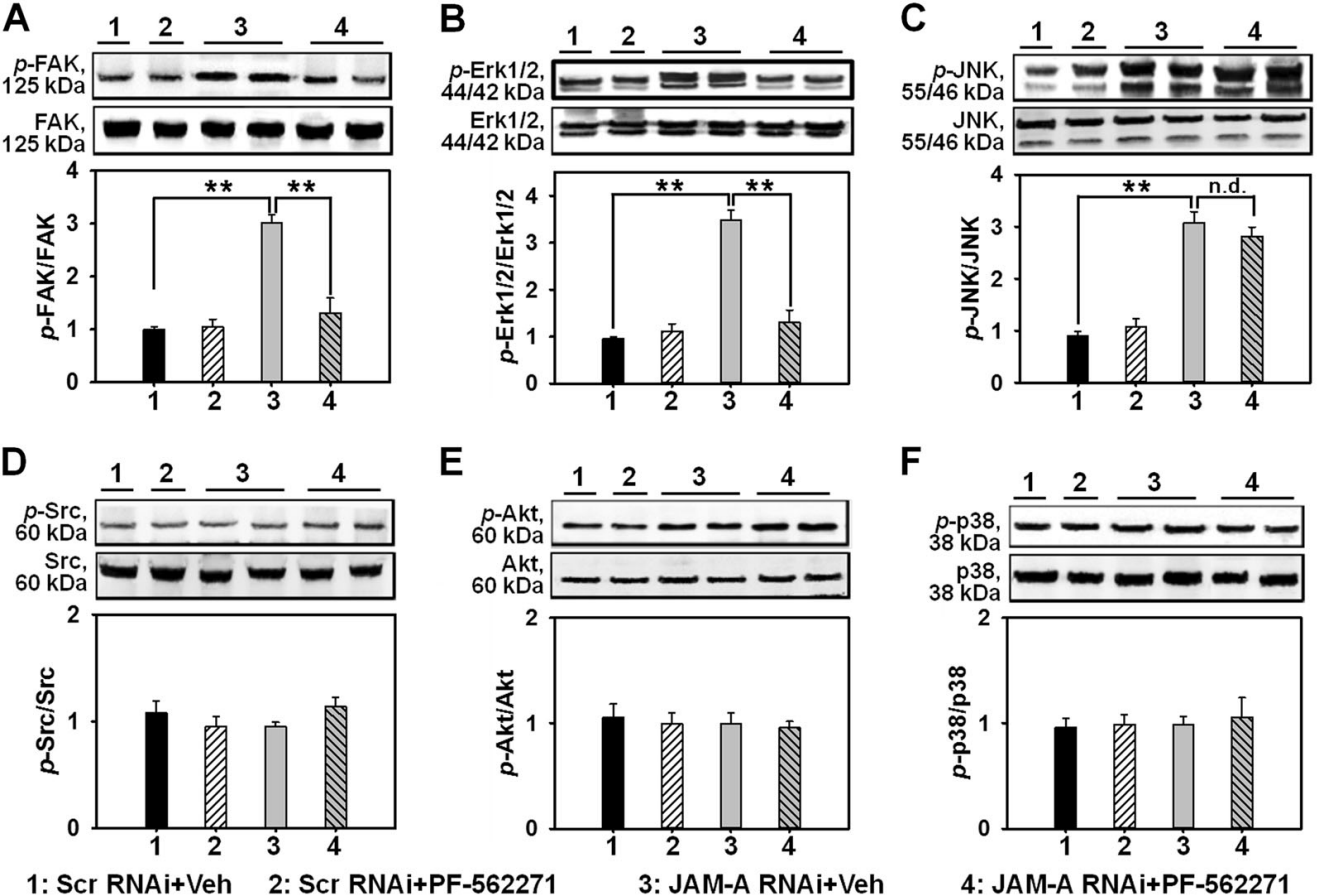
Effect of PF-562271 pre-administration on the expression of selected signaling molecules after JAM-A silencing in keratinocytes. Keratinocytes received 2-h pre-incubation with PF-562271 or DMSO (as vehicle control, Veh) followed by JAM-A RNAi. Immunoblotting was performed to investigate the protein level change of the total FAK (**a**), Erk1/2 (**b**), JNK (**c**), Src (**d**), Akt (**e**), and p38 (**f**), also the corresponding phosphorylated molecules. Densitometric analysis on the levels of phosphorylated proteins was normalized against the corresponding total protein levels which was then designated as 1. Bars are the means ± SD, *n* = 4. **P < 0.01; n.d. not detectable. ‘‘1’’, Scr RNAi + Veh; ‘‘2’’, Scr RNAi + PF-562271; ‘‘3’’, JAM-A RNAi + Veh; ‘‘4’’, JAM-A RNAi + PF-562271

Next we used PF-562271, an FAK putative inhibitor, to examine any change on the expression of above-mentioned signaling molecules. Immunoblots showed that PF-562271 evidently prevented the increase of *p*-FAK level induced by JAM-A knockdown (Fig. 4a), indicating that PF-562271 is a suitable inhibitor for FAK used herein. Next, we explored if PF-562271-induced FAK inactivation could interfere MAPK signaling. Data showed PF-562271 significantly inhibited the increase of *p*-Erk1/2 (Fig. 4b), however, the expression of *p*-JNK was not affected (Fig. 4c). Moreover, the expression of *p*-Src, *p*-Akt, and *p*-p38 (Fig. 4d, f) maintained unchanged after JAM-A knockdown or PF-562271 administration. These data indicate that the activation of FAK triggers Erk1/2 MAPK while not JNK after JAM-A silencing.

### Inhibiting FAK or Erk1/2 destroyed the expedited keratinocyte migration and proliferation induced by JAM-A knockdown

**Fig. 5:**
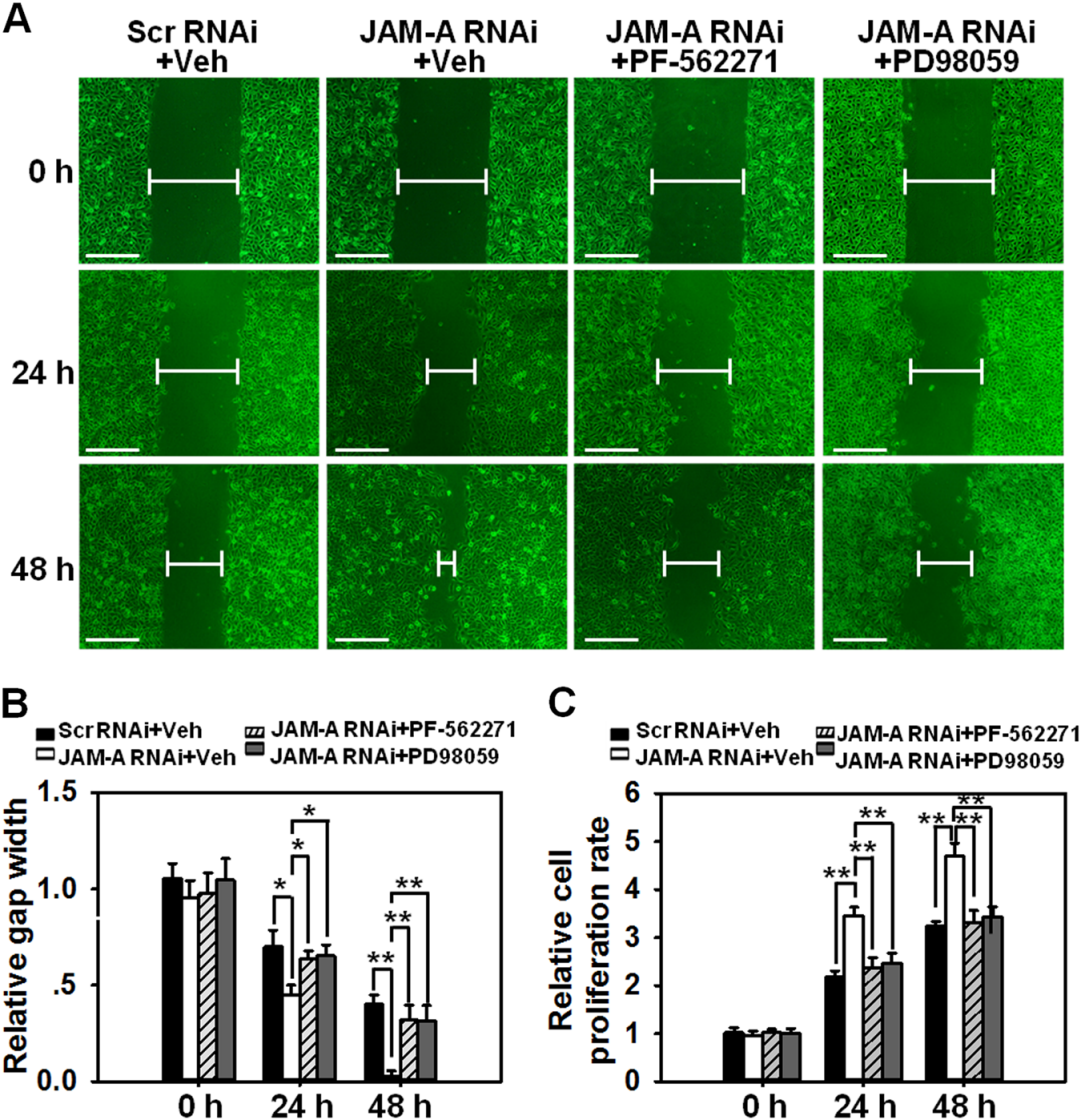
Effect of PF-562271 or PD98059 pre-administration on keratinocyte proliferation and migration after JAM-A silencing. **a** Keratinocytes received 2-h pre-incubation with PF-562271, PD98059, or DMSO followed by JAM-A RNAi. In addition, mitomycin C was used to block the proliferation prior to the scratch. Images at 0, 24, and 48 h post-scratching were captured to display the process of gap closure. Scale bars = 100 µm. **b** Densitometric analysis of the data from scratch assay normalized against the gap length at 0 h in scramble RNAi group which was designated as 1. Bars are means ± SD, *n* *=* 4. *P < 0.05; **P < 0.01. **c** The proliferation of keratinocytes after JAM-A silencing and drug administration was evaluated by MTT assay at 0, 24, and 48 h. Bars represent the mean ± SD, *n* = 4. **P < 0.01

To further prove the involvement of FAK and Erk1/2 in JAM-A silencing-mediated enhancement of cell proliferation and migration, related experiments were conducted with the use of PF-562271 and/or PD98059. Data showed that PF-562271 or PD98059 provoked the notable delay of scratch gap closure at 24 h and 48 h post drug treatment (Fig. 5a, b) and the deceleration of cell proliferation at 24 h and 48 h post-treatment (Fig. 5c) in JAM-A RNAi-transfected keratinocytes compared to Scr RNAi control. These data further confirm the participation of FAK and Erk1/2 in JAM-A-mediated keratinocyte migration and proliferation.

### In vivo knockdown of JAM-A promotes wound healing of the dorsal skin in adult rat

**Fig. 6:**
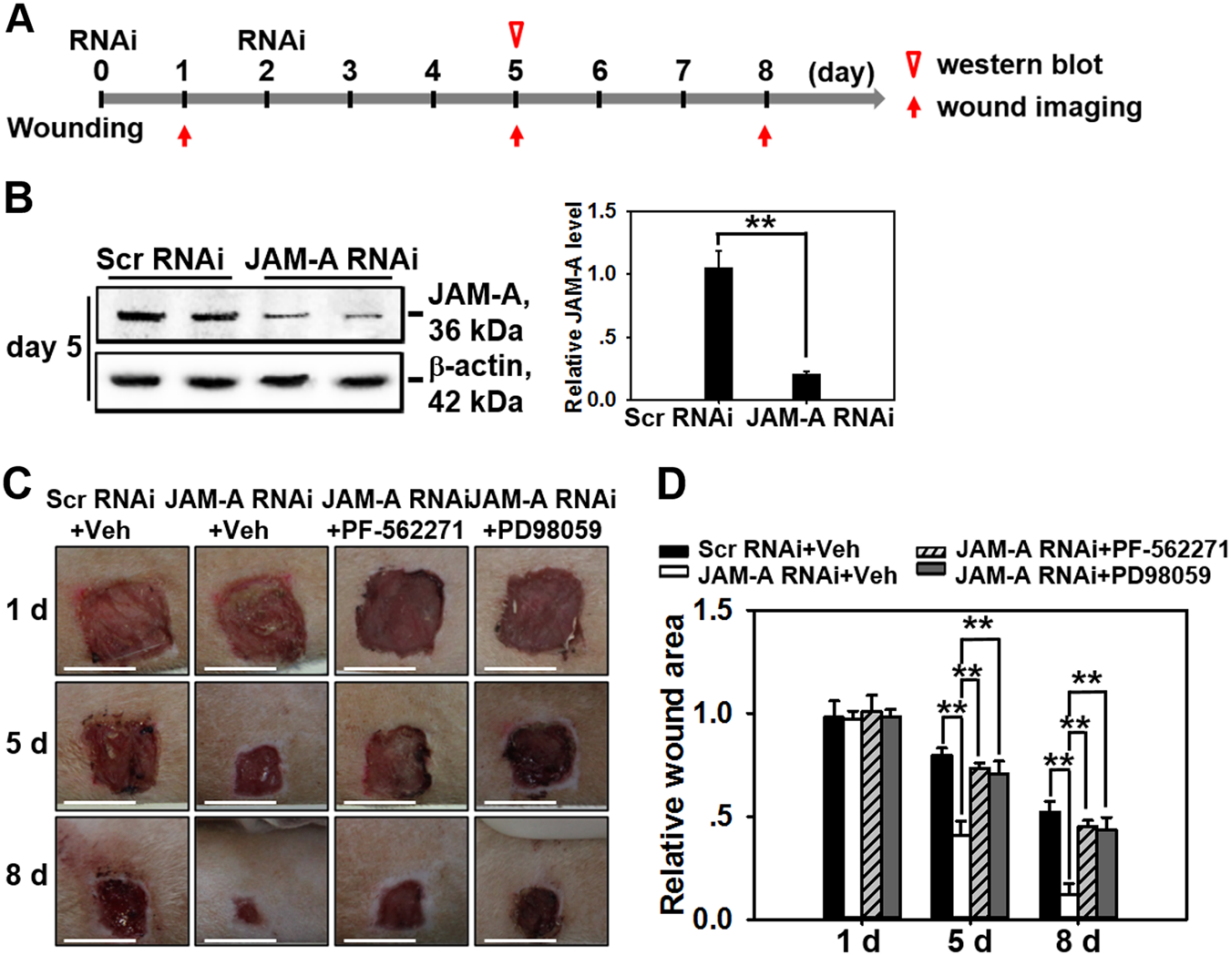
Study to assess the effect of JAM-A knockdown on wound healing in vivo. **a** Time points selected for the in vivo study. Generally, on day 0 the dorsal skin of SD rats received 1 × 1 cm^2^ full-thickness excision followed by immediate RNAi transfection at the wound edge, the RNAi transfection was repeated one more time on day 2. **b** Skin tissues at the wound edge from day-5 rats were collected, followed by western blot analysis. Densitometric analysis of JAM-A immunoblotting data normalized against actin with scramble RNAi arbitrarily set at 1. Each bar represents mean ± SD from four experiments. **P < 0.01. **c** In wound healing assay, DMSO, PF-562271, or PD98059 was co-administrated with siRNA on day 0 and 2. Images showing the healing process of rat dorsal skin wounds were captured and recorded on day 1, 5, and 8. Scale bars = 1 cm. **d** Densitometric analysis of the in vivo wound healing data normalized against the wound area on day 1 in (Scr RNAi + Veh) group which was designated as 1. Each bar represents the mean ± SD, *n* = 4. **P < 0.01

The main aim of this study was to investigate whether JAM-A knockdown could improve the healing process in animals or finally in humans, if so, whether the process is regulated through FAK-Erk1/2 pathway as indicated in above cell experiments. Adult SD rats received 1 × 1 cm^2^ incision on dorsal skin when was designated as day 0, followed by JAM-A RNAi administration at wound edge in epidermis of the skin on day 0 (immediately after wounding) and day 2, followed by western blot and wound imaging at designated time points as shown in Fig. 6a. On day 5 post-wounding, skins receiving RNAi administration were harvested and subjected to western blot analysis, which exhibited 80% shrinkage of JAM-A protein level in JAM-A RNAi-treated samples compared to control (Fig. 6b). Immunocytofluorescent staining of JAM-A in rat keratinocytes isolated from wound edge skin tissues on day 5 further confirmed the efficiency of JAM-A knockdown by RNAi in vivo (Supplementary Fig. S5). Moreover, *p*-FAK and *p*-Erk1/2 were activated after JAM-A knockdown in vivo, while when rats were pre-treated with PF-562271, JAM-A knockdown did not induce the activation of *p*-FAK and *p*-Erk1/2 as demonstrated by immunocytofluorescence analysis of *p*-FAK (Supplementary Fig. S6) and *p*-Erk1/2 (Supplementary Fig. S7) in rat keratinocytes isolated from wound edge skin tissues, which was in accordance with the in vitro data in Fig. 4. In order to evidently evaluate results from JAM-A silencing in vivo, images of the injured sites on rat back skin under different treatments were continuously monitored. It should be noted that PF-562271 or PD98059 was co-administrated with siRNA duplexes on day 0 and day 2. Results showed that JAM-A knockdown remarkably helped wound closure from day 5 to day 8, while co-administration of JAM-A RNAi with PF-562271 or PD98059 significantly delayed the healing process (Fig.6c, d). These data suggest that JAM-A knockdown in vivo would accelerate the wound healing process of rat skin possibly through FAK-Erk1/2 signaling.

## Discussion

JAM-A is broadly recognized as a structural protein and cell adhesion molecule at intercellular tight junction in epithelial and endothelial cells^22^, however, accumulating evidences suggest that JAM-A’s function in cellular physiology is far beyond that mentioned above. For instance, the colony formation, migration, and proliferation of multiple myeloma were found to be damaged when JAM-A was inhibited in vitro^23^. Also, tumor development was shown to be impeded by JAM-A antibody in a multiple myeloma model with murine xenograft^23^, suggesting JAM-A as a prognostic factor and new therapeutic target in multiple myeloma.

In the current study, JAM-A was for the first time found to negatively regulate the proliferation and migration of primary human keratinocytes, as well as in vivo cutaneous wound healing in adult rats, the regulatory mechanism was partially illustrated. Our results showed that JAM-A predominantly expressed at cell–cell interface in the epidermis of normal human skin (Fig. 1), confirming its involvement as a putative junctional molecule. Knockdown of JAM-A by RNAi accelerated keratinocyte proliferation and migration (Fig. 2), combined with a significant activation of FAK, Erk1/2, and JNK (Fig. 3), suggesting the possible involvement of FAK, Erk1/2, or JNK signaling in JAM-A RNAi-induced enhancement of keratinocyte proliferation and migration. Inhibition of FAK by PF-562271 abolished the phosphorylation of Erk1/2 but not JNK (Fig. 4), suggesting that FAK may not be essential for JNK activation. FAK inhibition by PF-562271 and Erk1/2 inhibition by PD98059 also caused the reduced movement and proliferation in keratinocytes (Fig. 5a–c), indicating that JAM-A knockdown-induced Erk1/2 activation is FAK-dependent, which further regulated the enhancement of keratinocyte migration and proliferation. More crucially, in vivo JAM-A knockdown (Fig. 6a, b) on rat dorsal skins surrounding the wound areas remarkably accelerated the healing process, while the use of PF-562271 or PD98059 obviously abolished this effect (Fig. 6c, d). These findings overall suggest that loss of JAM-A promotes keratinocyte proliferation and migration, and the in vivo healing course, which is regulated by the signaling of FAK-mediated Erk1/2 activation. It is worthy to note that although the half-life of PF-562271 or PD98059 is not specified by the manufacturer, the working concentration and treatment duration regarding these two drugs are selected with great caution based on several important literatures^16,17,24,25^ and our pilot experiments. The optimum concentration and treatment duration of JAM-A siRNA duplexes were also carefully selected based on our previously published works^26^ and preliminary data, we found that transfection of keratinocytes with 100 nM JAM-A siRNA duplex (50 nM seq#1 + 50 nM seq#2, see Supplemental Fig. S3) for 24 h (Supplemental Fig. S4) could obtain the optimal knockdown effect on JAM-A level.

FAK is a non-receptor tyrosine kinase with a molecular weight of 125 kDa. FAK participates in the formation of focal adhesion between cell and extracellular matrix, it mediates integrin-involved signal transduction pathway. It was found that when FAK activity was inhibited, breast cancer lost its metastatic ability due to reduced mobility^27^. It can be activated at Tyr397 residue via phosphorylation^28^. Using anti-*p*-FAK^[Y397]^, we examined FAK activation in primary human keratinocytes following JAM-A knockdown and found that JAM-A knockdown significantly stimulated FAK activity. It is noteworthy that the phosphorylation of FAK, which leads to its activation usually takes place within minutes^29^, therefore, keratinocytes were treated with PF-562271 in advance to prevent any FAK signaling. We found that PF-562271 blocked the phosphorylation of FAK, and suppressed the proliferation and migration of keratinocytes, indicating the involvement of FAK in JAM-A knockdown-mediated acceleration of cell proliferation and migration.

The activation of FAK and MAPK is required for migration^4^. It is known that the activation of Erk is essential for endothelial cell migration during revascularization^30^. Erk1/2 phosphorylation and activation were observed in the process of skin re-epithelialization^31^. Also, Erk1/2 phosphorylation was found to regulate formononetin-involved regeneration of endothelium^32^. Moreover, when Erk signaling was inhibited, the wound healing process was found to be completely impeded^33^, these conclusions are consistent with results from the current study that the use of PD98059 to block Erk signaling remarkably delayed the gap closure rate in keratinocyte cultures. Accumulating studies are linking the accelerated cell proliferation and migration with MAPK activation. Herein, Erk1/2 was found to exert a positive influence on the migration and proliferation of primary keratinocytes after JAM-A silencing.

MAPKs are known to participate in many cellular responses and regulate multiple cellular behaviors, such as cell survival, proliferation, apoptosis, migration, and gene expression. However, it is unknown how MAPKs are affected by other cross-talking signaling during the healing process. MAPKs have been found as one of the predominant downstream targets of FAK-mediated cascades^34,35^. Accordingly, the current study has demonstrated that JAM-A silencing-induced phosphorylation and activation of Erk1/2 signaling was FAK-dependent, nevertheless, the activation of JNK or p38 MAPKs was clearly FAK-independent following JAM-A knockdown. MTT assay and cell scratch assay in primary keratinocyte cultures further illustrated the involvement of MAKPs in certain cellular behaviors, data showed that the migration and proliferation of keratinocytes were regulated via Erk1/2 signaling instead of JNK or p38 MAPKs.

In conclusion, this work demonstrates that FAK phosphorylation induced by JAM-A knockdown triggered the activation of Erk1/2 signaling, which further promoted keratinocyte proliferation and migration, and significantly improved the skin healing process in vivo. More investigations would be required to reveal underlying mechanisms how JAM-A RNAi causes the remarkable surge on FAK activity. Experiments with JAM-A overexpression using both primary human keratinocytes and in vivo rat dorsal skins are intensively moving onto further verify the conclusion in this study from a different view. Finally, the insights into JAM-A-FAK-Erk1/2 signaling are helpful for us to develop pharmacological interventions that would be beneficial for treating chronic wounds.

## Materials and methods

### Antibodies

Primary antibodies were purchased from different vendors listed in Supplementary Table S2. Each one used herein was pre-screened by western blot utilizing lysates from normal human skins and keratinocytes at various protein concentrations. Each antibody was confirmed to specifically crossreact with its target antigen at the expected molecular weight.

### Keratinocyte isolation and culture

Primary human keratinocytes were isolated as previously described^18^. Briefly, the whole skin specimens were washed with phosphate-buffered saline (PBS) to remove all contaminated bloods. Subcutaneous fats were shaved off manually by forceps and scissors, the rest samples were sheared into small pieces, which were further seeded onto the culture plates containing 0.25% dispase II (Roche, Mannheim, Germany) and incubated for at 4 °C overnight. On the following day, epidermal tissues were separated from dermal tissues by using forceps and then placed onto 6-mm culture dishes containing 0.25% trypsin (Hyclone, Little Chalfont, UK) for 10 min. Next, the epidermal samples were further dissected into smaller pieces with forceps. The suspension was then briefly spinned down, keratinocytes were precipitated, resuspended, and cultured in KGM-Gold media.

### Transfection of keratinocytes by RNAi in vitro

Keratinocyte cultures with an ~80% confluence received scramble or JAM-A-specific RNAi at the working concentration of 100 nM siRNA duplexes in each transfection system. To silence JAM-A, a mixture of 100 nM JAM-A siRNA duplexes (50 nM sequence #1: 5′-GGAUAGUGAUGCCUACGAAdTdT-3′ + 50 nM sequence #2: 5′-dTdTCCUAUCACUACGGAUGCUU-3′, RiboBio Co. Ltd., Guangzhou, China) vs. 100 nM nontargeting scramble siRNA duplexes was used. Lipofectamine 2000 was used as the transfection reagent. The transfection reaction was terminated 24 h later by discarding the media containing siRNA and Lipofectamine 2000. Keratinocytes were rinsed with PBS three times and incubated in new KGM-Gold media.

### Drug treatment in primary keratinocytes

Keratinocytes received a 2-h pre-incubation with PF-562271 at the working concentration of 10 µM (Selleck Chemicals)^16^, or PD98059 at the working concentration of 10 µM (Aladdin Co., Ltd, Shanghai, China)^17^, prior to JAM-A knockdown to block the potential FAK and Erk1/2 signaling. PF-562271 and PD98059 were dissolved in DMSO, which served as the vehicle control in this study.

### **S**cratch assay

In vitro scratch assay was performed in 24-well plates. Primary keratinocytes were cultured at 37 °C to reach a 90% confluence. Scratch was created in each well by using a sterile 200-μl pipette tip. Notably, cells received 1-h mitomycin C treatment to block the proliferation prior to scratching. After scratching, keratinocytes were grown in fresh KGM-Gold medium at 37 °C. Images of the scratch gap were captured and recorded at 0, 24, and 48 h under phase contrast microscope, and analyzed by Image-Pro Plus software. All scratch coverages were measured in quadruplicate at four different sites.

### MTT assay

The proliferation of primary keratinocytes was evaluated by 3–4, 5-dimethylthiazol-2-yl-2, 5-diphenyl-tetrazolium bromide (MTT) assay, which was conducted in 96-well plates. Keratinocytes first received a 4-h MTT treatment at the concentration of 0.1 mg/ml, then lysed in DMSO for 10 min. The OD value at 560 nm obtained from each well was measured and recorded by a microplate reader (Bio-Rad, Hercules, CA) at designated time points of 0, 24, and 48 h 2 days after transfection.

### In vivo wound healing assay

To create the full-thickness skin wounds, adult SD rats received intramuscular injection of ketamine (50 mg/kg b.w.) and xylazine (5 mg/kg b.w.), which were commonly used as anesthetics to relief pain and suffering. Skins at dorsum region were then shaved by clippers and completely sterilized with 70% alcohol. Under aseptic condition, 1.0 × 1.0 cm^2^ square incisions with full-thickness skin layer were made in rat dorsum region by using disposable blade, when was marked as day 0. Immediately after wounding, JAM-A-specific or scramble siRNAs at 100 nM were topically injected into skins at the wound edge. siRNA injection was repeated one more time on day 2. Drug treatment with PF-562271 (10 µM) or PD98059 (10 µM) was pursued simultaneously with siRNAs. Images displaying the in vivo healing process were captured and recorded on day 1, day 5, and day 8 by Canon digital camera.

### Western blot analysis

Normal foreskin samples from six patients were collected in 500 µl of ice-cold IP lysis buffer containing 1% NP-40, which was freshly replenished with protease and phosphatase inhibitors (Sigma-Aldrich). Samples then received ultrasonic decomposition followed by centrifugation at 13,000 × *g* at 4 °C for 40 min to obtain the supernatants. Protein concentration was determined by spectrophotometry using a Bio-Rad Model 680 Plate Reader. Next, 30 µg total proteins per lane were separated by SDS-PAGE using gels of different concentrations for different target proteins, and then transferred onto nitrocellulose membranes. The non-specific binding sites on membrane were blocked with 5% non-fat milk at room temperature (RT) for 1 h. Primary antibodies at corresponding working dilution (Supplementary Table S2) were applied into the chamber keeping membranes at 4 °C overnight. The next day, membranes were rinsed with PBS-Tris and applied with HRP-conjugated secondary antibodies. Immunoreactive signals were detected by the enhanced chemiluminescent reaction. Images were acquired with a LAS-4000 luminescent image analyzer (FujiFilm).

### Immunohistochemistry

Paraffin-embedded human foreskin sections at 4-μm thickness were obtained by using a cryostat machine at −20 °C. Sections were fixed with 4% paraformaldehyde (w/v) for 15 min, followed by permeabilization with 0.1% Triton X-100 for 10 min at room temperature. Then sections were incubated with anti-JAM-A antibody with the dilution of 1:50 at 4 °C overnight. The next day, sections were incubated with HRP-streptavidin conjugated secondary antibody for 30 min. DAB Detection System kit (Millipore, Billerica, MA) was then utilized to observe the brownish JAM-A precipitations. Cell nuclei were counterstained with hematoxylin.

### Quantitative real-time PCR

Total RNA was extracted from homogenized primary keratinocytes by using Total RNA Isolation Kit (Takara, Japan). The RNA purity was evaluated by A260: A280 ratio. In brief, 2.0 µg RNA sample was reversely transcribed by using PrimeScript™ RT Reagent Kit (Takara, Japan). The obtained cDNA was amplified with SYBR® Premix Ex Taq™ Kit (Takara, Japan), specific primers for each gene were listed in Supplementary Table S1. The mRNA level of each gene was finally normalized against GAPDH mRNA level. The thermal cycle condition was as follows: initial denaturation at 95 °C for 30 s, denaturation at 95 °C for 15 s, annealing at 60 °C for 30 s, elongation at 72 °C for 10 s for 40 cycles. Upon the completion of the reaction, a melting curve analysis (65–105 °C) was conducted to check if primer dimmers exist. The relative concentration of each target gene was determined by cycle threshold (Ct) at which specific fluorescence signal became detectable. Ct value was used for kinetic analysis and was proportional to the initial number of target copies in the sample. Real-time PCR data was finally exported and processed using the ∆∆CT method.

### Statistical analysis

SPSS version 21 (SPSS Inc, Chicago, Ill) was employed for the statistical analysis, one-way ANOVA followed by Dunnett’s test was used as appropriate. Data were presented as mean ± standard deviation (SD). Each experiment was repeated using samples from at least four individuals. *P* *<* 0.05 was seen as statistically significant.

## Electronic supplementary material

Supplemental Materials
