# Supplementary material for: JAM-A knockdown accelerates the proliferation and migration of human keratinocytes, and improves wound healing in rats via FAK/Erk signaling
Source: Cell Death Dis. 2018 Aug 28;9(9):848. doi: 10.1038/s41419-018-0941-y (PMC6113279; doi:10.1038/s41419-018-0941-y)
Supplement: Supplementary file 1 — Supplemental Materials [file 41419_2018_941_MOESM1_ESM.docx]

**Supplemental Materials**

**JAM-A knockdown accelerates the proliferation and migration of human keratinocytes, and improves wound healing in rats *via* FAK/Erk signaling**

Yunchuan Wang^1, 2, #^, Jianping Zheng^3, #^, Yue Han^4, #^, Yijie Zhang^2^, Linlin Su^2^, Dahai Hu^2^, Xiaobing Fu^1^

^1^Institute of Basic Medicine, Chinese PLA General Hospital, Beijing 100853, P.R. China

^2^Department of Burns and Cutaneous Surgery, Xijing Hospital, the Fourth Military Medical University, Xi’an, Shaanxi 710032, P.R. China

^3^Department of Orthopedic Surgery, Xiangyang Central Hospital, the Affiliated Hospital of Hubei University of Arts and Science, Xiangyang, Hubei 441021, P.R. China

^4^Department of Burns and Plastic Surgery, Xi’an Central Hospital, Xi’an, Shaanxi710003, P.R. China

^#^The three authors contributed equally to this work.

Correspondence should be addressed to:

Xiaobing Fu, [fuxiaobing@vip.sina.com](mailto:fuxiaobing@vip.sina.com). Institute of Basic Medicine, Chinese PLA General Hospital, Beijing 100853, P.R. China.

**Short Title**

JAM-A knockdown promotes wound healing via FAK/Erk

**Key Words**

Junctional adhesion molecule-A, keratinocytes, proliferation, migration, wound healing

**
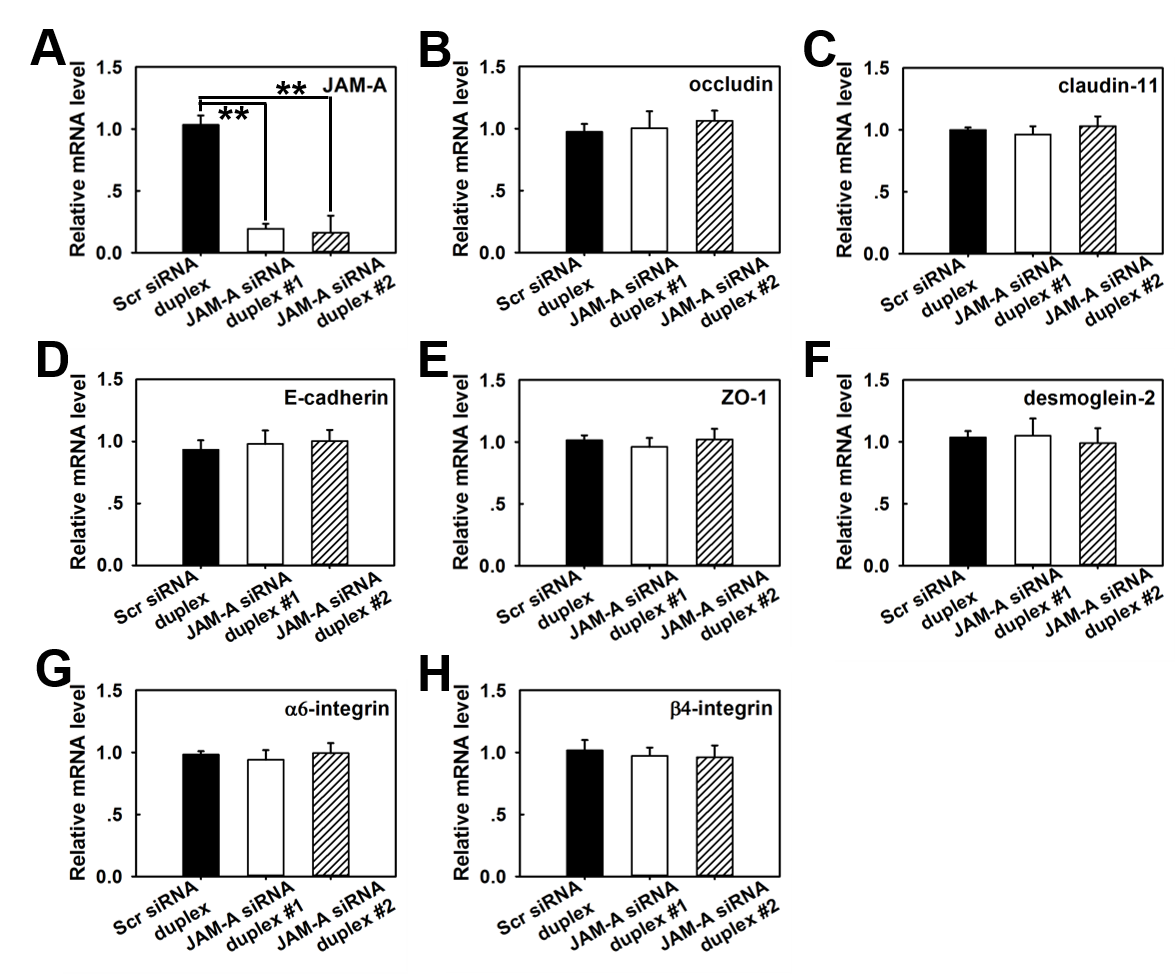
**

**Fig. S1. Effect of JAM-A knockdown by RNAi on mRNA levels of selected cell junction proteins in primary human keratinocytes.** Two different JAM-A siRNA duplexes (#1 and #2) at 100 nM were used to individually suppress JAM-A level compared with the 100 nM scramble siRNA duplex. The mRNA levels of JAM-A (a tight junction protein, ***A***), occludin (a tight junction protein, ***B***), claudin-11(a tight junction protein, ***C***), E-cadherin (an adhesion junction protein, ***D***), ZO-1 (a tight junction adaptor, ***E***), desmoglein-2 (a desmosome junction protein, ***F***), α6-integrin (a hemi-desmosome junction protein, ***G***), and β4-integrin (a hemi-desmosome junction protein, ***H***) were examined by quantitative real-time PCR at 24 h after the completion of 24 h-siRNA transfection. The mRNA level of JAM-A was specifically inhibited by either JAM-A siRNA duplex, but not other examined molecules. The mRNA level of each protein in scramble siRNA-treated cells was arbitrarily set at 1. Each bar represents mean ± SD of three to four independent experiments. ***P* < 0.01.


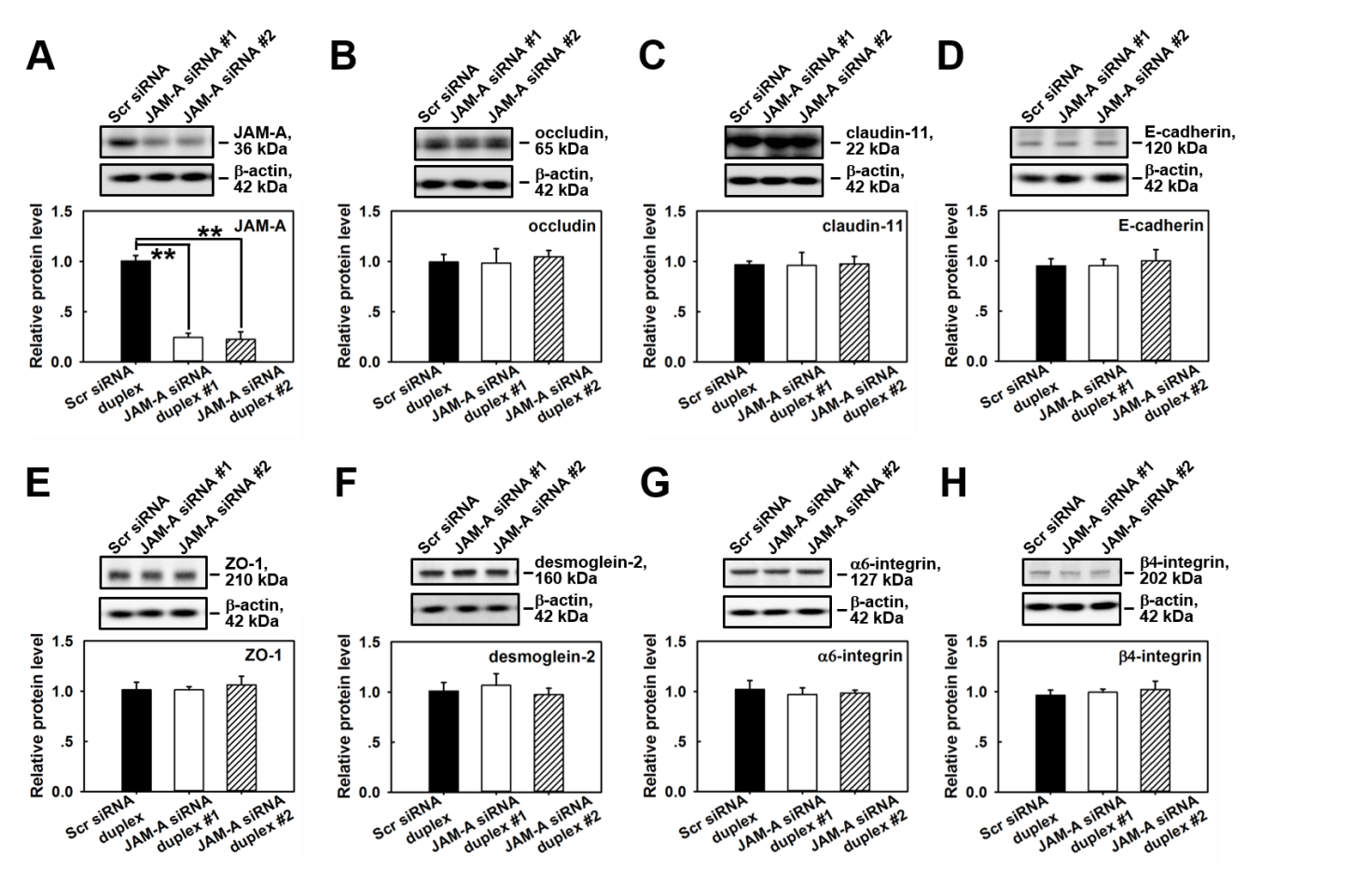


**Fig. S2. Effect of JAM-A knockdown by RNAi on protein levels of selected cell junction proteins in primary human keratinocytes.** Two different JAM-A siRNA duplexes (#1 and #2) at 100 nM were used to individually suppress JAM-A level compared with the 100 nM scramble siRNA duplex. The protein levels of JAM-A (***A***), occludin (***B***), claudin-11 (***C***), E-cadherin (***D***), ZO-1 (***E***), desmoglein-2 (***F***), α6-integrin (***G***), and β4-integrin (***H***) were examined by western blot at 48 h after the completion of 24 h-siRNA transfection. The protein level of JAM-A was specifically suppressed by either JAM-A siRNA duplex, but not other examined molecules. Each immunoblot was normalized against its corresponding actin with the value in scramble siRNA group arbitrarily set at 1. Each bar represents mean ± SD of three to four independent experiments. ***P* < 0.01.


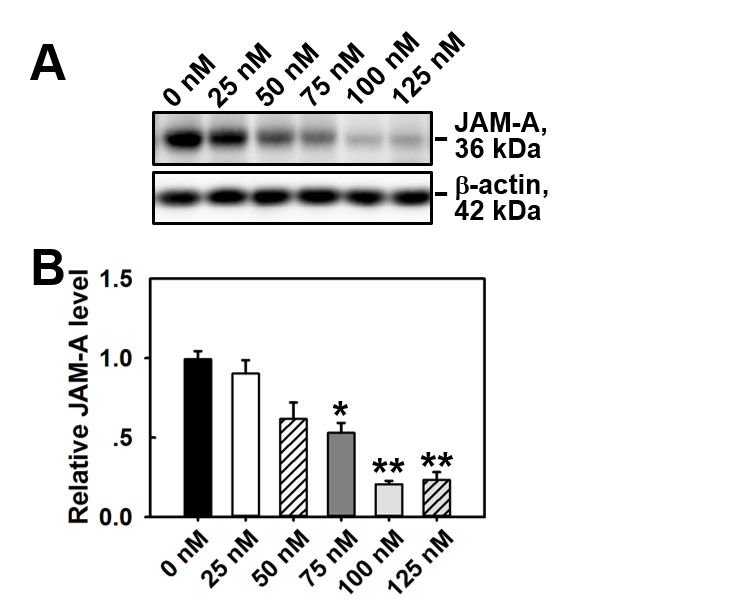


**Fig. S3. The knockdown efficiency of JAM-A siRNA duplex at different concentrations in primary human keratinocytes.** (***A***) Immunoblots showing the protein level of JAM-A after treating keratinocytes with the specific JAM-A siRNA duplexes at different concentrations of 0 nM (0 nM JAM-A siRNA + 125 nM scramble siRNA) , 25 nM (12.5 nM JAM-A siRNA sequence #1 + 12.5 nM JAM-A siRNA sequence #2 + 100 nM scramble siRNA), 50 nM (25 nM seq#1 + 25 nM seq#2 + 75 nM scr seq), 75 nM (37.5 nM seq#1 + 37.5 nM seq#2 + 50 nM scr seq), 100 nM (50 nM seq#1 + 50 nM seq#2 + 25 nM scr seq), and 125 nM (62.5 nM seq#1 + 62.5 nM seq#2 + 0 nM scr seq). (***B***) Densitometric analysis of JAM-A immunoblotting data normalized against corresponding β-actin level with the value in 0 nM group arbitrarily set at 1. Each bar represents mean ± SD of three independent experiments. **P* < 0.05; ***P* < 0.01.


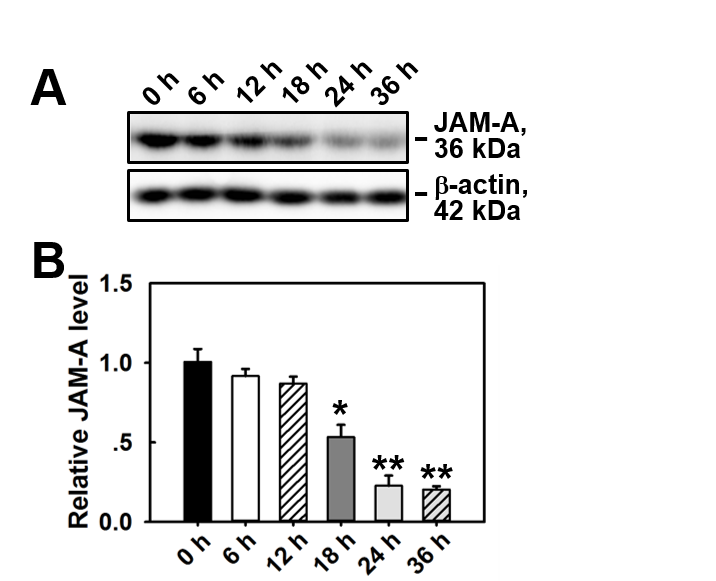


**Fig. S4. The knockdown efficiency of JAM-A siRNA duplex for different treatment duration in primary human keratinocytes.** (***A***) Immunoblots showing the protein level of JAM-A after transfecting keratinocytes with 100 nM JAM-A siRNA duplexes (50 nM seq#1 + 50 nM seq#2) for different treatment duration of 0 h, 6 h, 12 h, 18 h, 24 h, and 36 h. (***B***) Densitometric analysis of JAM-A immunoblotting data normalized against corresponding β-actin level with the value in 0 h group arbitrarily set at 1. Each bar represents mean ± SD of three independent experiments. **P* < 0.05; ***P* < 0.01.


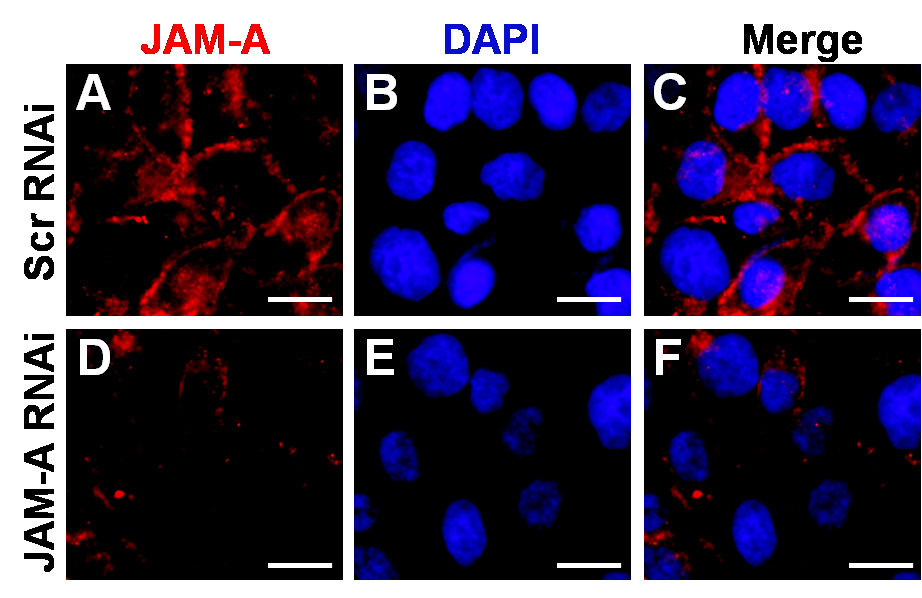


**Fig. S5. Immunocytofluorescent staining to assess the efficiency of JAM-A knockdown in primary rat keratinocytes.** Skin tissues at the wound edge from day-5 rats that received the scramble or JAM-A-specific siRNA duplexes were collected, keratinocytes were isolated, cultured, and stained for immunocytofluorescent analysis. JAM-A was stained red (***A***, ***D***), nuclei were stained with DAPI (*blue*, ***B***, ***E***), the merged images were shown in (***C***, ***F***). Scale bar: 20 µm. ‘Scr’, scramble.


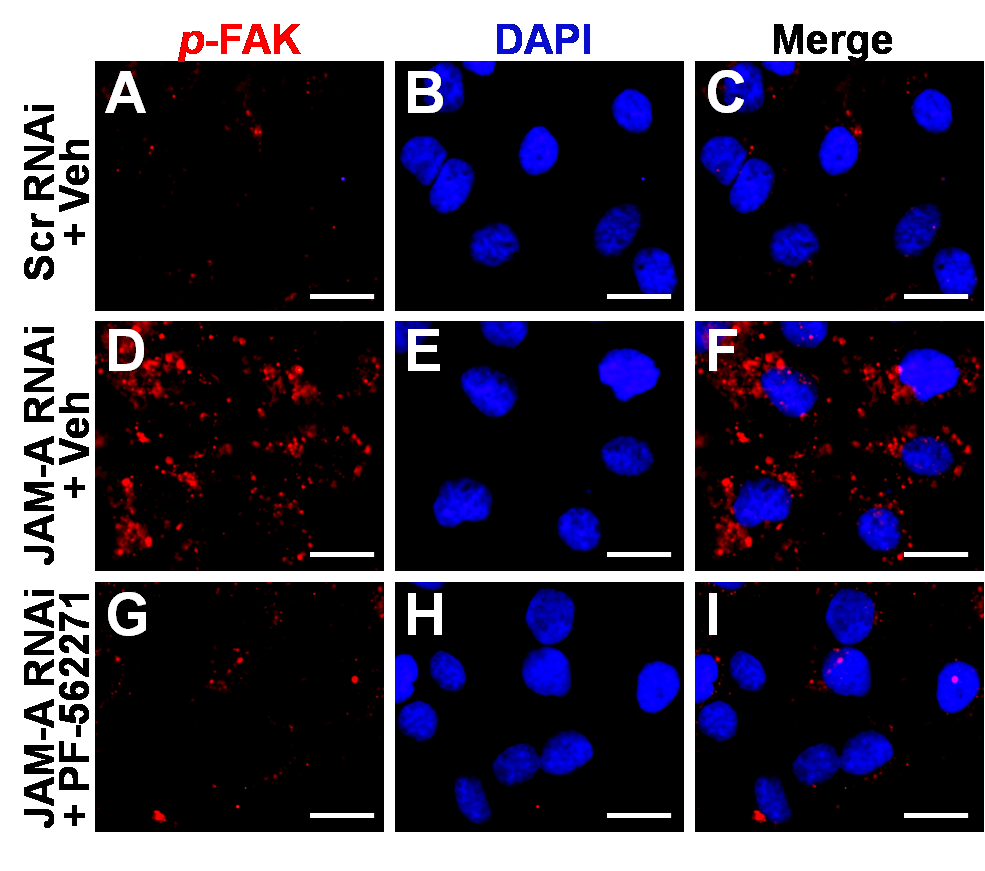


**Fig. S6. Immunocytofluorescent staining to assess the effect of PF-562271 on the expression and localization of *p*-FAK in rat keratinocytes isolated from the wound edge skin tissues that received RNAi.** Rat skin tissues at the wound edge received vehicle (DMSO) or PF-562271 pretreatment for 6 h followed by RNAi transfection with scramble or JAM-A-specific siRNA duplexes. Samples on day 5 were collected, rat keratinocytes were isolated, cultured, and stained for immunocytofluorescent analysis. *p*-FAK was stained red (***A***, ***D, G***), nuclei were stained with DAPI (*blue*, ***B***, ***E, H***), the merged images were shown in (***C***, ***F, I***). Scale bar: 20 µm. ‘Scr’, scramble. ‘Veh’, vehicle.


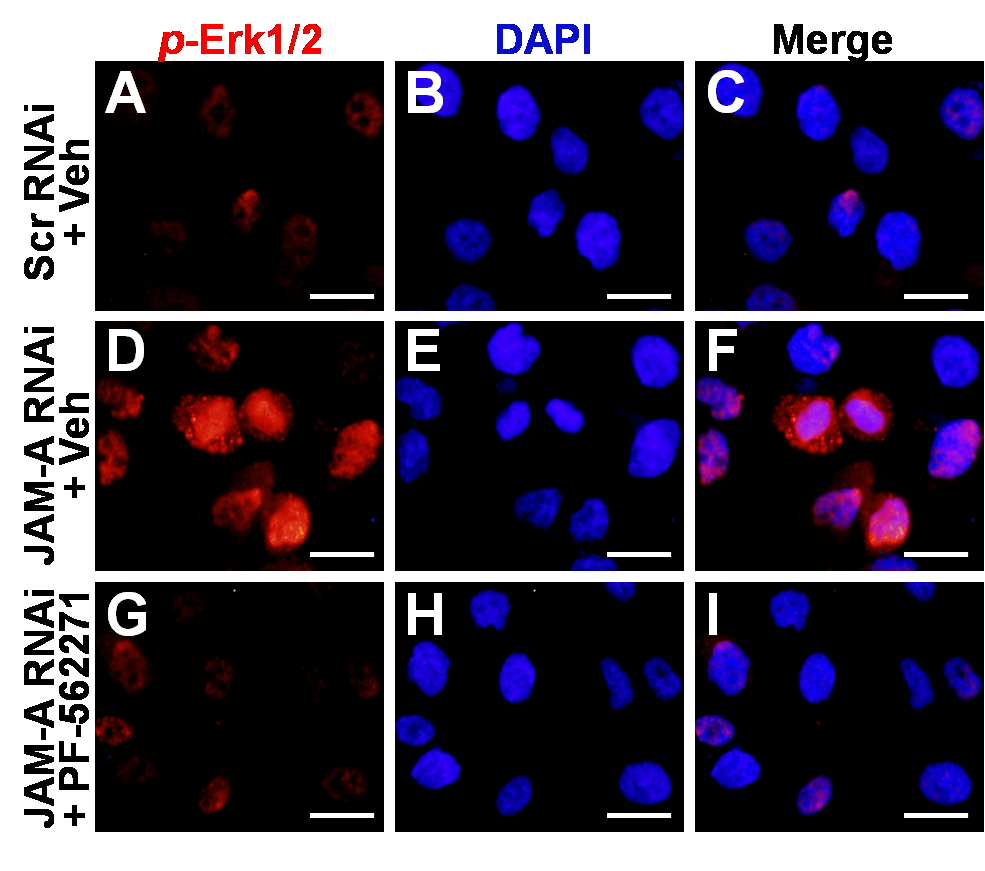


**Fig. S7. Immunocytofluorescent staining to assess the effect of PF-562271 on the expression and localization of *p*-ERK1/2 in rat keratinocytes isolated from the wound edge skin tissues that received RNAi.** Rat skin tissues at the wound edge received vehicle (DMSO) or PF-562271 pretreatment for 6 h followed by RNAi transfection with scramble or JAM-A-specific siRNA duplexes. Samples on day 5 were collected, rat keratinocytes were isolated, cultured, and stained for immunocytofluorescent analysis. *p*-Erk1/2 was stained red (***A***, ***D, G***), nuclei were stained with DAPI (*blue*, ***B***, ***E, H***), the merged images were shown in (***C***, ***F, I***). Scale bar: 20 µm. ‘Scr’, scramble. ‘Veh’, vehicle.

**Table S1. Primers used for quantitative real-time PCR in this study.**

| **Gene** | **Forward primer** | **Reverse primer** |
| --- | --- | --- |
| ***JAM-A*** | 5’- ATCTGGTTTGCCTATAGCCG -3’ | 5’- AGGAATGACGAGGTCTGTTT -3’ |
| ***occludin***  ***claudin-11***  ***E-cadherin***  ***ZO-1***  ***desmoglein-2***  ***α6-integrin***  ***β4-integrin***  ***GAPDH*** | 5’- TCAGGGAATATCCACCTATCACTTCAG -3’  5’- CGGTGTGGCTAAGTACAGGC -3’  5’- CGGGAATGCAGTTGAGGATC -3’  5’- CCCCACTCTGAAAATGAGGA -3’  5’- TGGACACCCAAACAGTGGCCCT -3’  5’- CAAGATGGCTACCCAGATAT -3’  5’- GGTCCAGGAAGATCCATTTCAA -3’  5’- CGAACTGTTTCACCAGCAAC -3’ | 5’- CATCAGCAGCAGCCATGTACTCTTCAC -3’  5’- CGCAGTGTAGTAGAAACGGTTTT -3’  5’- AGGATGGTGTAAGCGATGGC -3’  5’- GGGAACAACATACAGTGACGC -3’  5’- CTCACTTTGTTGCAGCAGCACAC -3’  5’- CTGAATCTGAGAGGGAACCA -3’  5’- TAGCAGACCTCGTAGGCTGTGA -3’  5’- GGTACATCTGGGGAACTTCT -3’ |

A.T., annealing temperature.

**Table S2. Antibodies used for different experiments in this study.**

|  |  |  |  | **Dilution** | |
| --- | --- | --- | --- | --- | --- |
| **Target protein** | **Catalog #** | **Host** | **Vendor** | **WB** | **IHC** |
| JAM-A | 36-1700 | Rabbit | Invitrogen | 1:250 | 1:50 |
| FAK | sc-558 | Rabbit | Santa cruz biotechnology | 1:200 |  |
| *p*-FAK^[Y397]^ | 8556 | Rabbit | Cell signaling technology | 1:1000 |  |
| c-Src | sc-8056 | Mouse | Santa cruz biotechnology | 1:200 |  |
| *p*-Src^[Y416]^ | 6943 | Rabbit | Cell signaling technology | 1:1000 |  |
| Akt | 4691 | Rabbit | Cell signaling technology | 1:1000 |  |
| *p*-Akt^[S473]^ | 4060 | Rabbit | Cell signaling technology | 1:1000 |  |
| Erk1/2 | 4695 | Rabbit | Cell signaling technology | 1:1000 |  |
| *p*-Erk1/2^[T202/Y204]^ | 4370 | Rabbit | Cell signaling technology | 1:2000 |  |
| JNK | 9258 | Rabbit | Cell signaling technology | 1:1000 |  |
| *p*-JNK^[T183/Y185]^ | 4668 | Rabbit | Cell signaling technology | 1:1000 |  |
| p38 | 8690 | Rabbit | Cell signaling technology | 1:1000 |  |
| *p*-p38^[T180/Y182]^ | 4511 | Rabbit | Cell signaling technology | 1:1000 |  |
| occludin | 71-1500 | Rabbit | Invitrogen | 1:250 |  |
| claudin-11 | 36-4500 | Rabbit | Invitrogen | 1:250 |  |
| E-cadherin  ZO-1 | sc-7870  61-7300 | Rabbit  Rabbit | Santa cruz biotechnology  Invitrogen | 1:200  1:250 |  |
| desmoglein-2  α6-integrin | sc-390531  ab235905 | Mouse  Rabbit | Santa cruz biotechnology  Abcam | 1:200  1:500 |  |
| β4-integrin | ab29042 | Mouse | Abcam | 1:500 |  |
| β-actin | sc-1616 | Goat | Santa cruz biotechnology | 1:200 |  |
